# The Importance of User Segmentation for Designing Digital Therapy for Adolescent Mental Health: Findings From Scoping Processes

**DOI:** 10.2196/12656

**Published:** 2019-05-08

**Authors:** Theresa Fleming, Sally Merry, Karolina Stasiak, Sarah Hopkins, Tony Patolo, Stacey Ruru, Manusiu Latu, Matthew Shepherd, Grant Christie, Felicity Goodyear-Smith

**Affiliations:** 1 Department of Psychological Medicine University of Auckland Auckland New Zealand; 2 Faculty of Health Victoria University of Wellington Wellington New Zealand; 3 Department of Social Work University of Auckland Auckland New Zealand; 4 Department of General Practice & Primary Health Care University of Auckland Auckland New Zealand

**Keywords:** gamification, computerized therapy, mental health, adolescent, mobile apps, internet

## Abstract

**Background:**

New Zealand youth, especially those of Māori and Pacific descent, have high rates of depression, anxiety, and self-harm, but have low rates of help-seeking from mental health professionals. Apps, computerized therapy, and other digital tools can be effective, highly scalable treatments for anxiety and depression. Co-design processes are often used to foster engagement with end users, but this does not always lead to high levels of engagement.

**Objective:**

We aimed to carry out preliminary scoping to understand adolescents’ current internet use and diversity of preferences to inform a planned co-design process for creating digital mental health tools for teenagers.

**Methods:**

Interactive workshops and focus groups were held with young people. Data were analyzed using a general inductive approach.

**Results:**

Participants (N=58) engaged in 2 whānau (extended family) focus groups (n=4 and n=5), 2 school- or community-based focus groups (n=9 each), and 2 workshops (n=11 and n=20). The authors identified 3 overarching themes: (1) Digital mental health tools are unlikely to be successful if they rely solely on youth help-seeking. (2) A single approach is unlikely to appeal to all. Participants had diverse, noncompatible preferences in terms of *look or feel* of an app or digital tool. The authors identified 4 user groups *players or gamers*, *engagers*, *sceptics*, and *straight-talkers*. These groups differed by age and degree of current mental health need and preferred gamified or fun approaches, were open to a range of approaches, were generally disinterested, or preferred direct-to-the-point, serious approaches, respectively. (3) Digital mental health tools should provide an immediate response to a range of different issues and challenges that a young person may face.

**Conclusions:**

Defining the preferences of different groups of users may be important for increasing engagement with digital therapies even within specific population and mental health–need groups. This study demonstrates the importance of scoping possible user needs to inform design processes.

## Introduction

### Background

Depression and anxiety, including clinical disorders and subclinical symptoms, are common among young people. New Zealand youth have high rates of mental distress, with rates particularly high among Māori and Pacific girls [1,2]. Most young people do not seek professional help for mental health needs [3], and this *forgone care* is particularly high among indigenous and minority youth [1,4,5].

Digital mental health tools have been shown to be effective and appealing treatments for anxiety and depression [6-8]. This has been demonstrated among different cultural and demographic groups, including Māori and Pacific youth [9,10]. By making interventions available conveniently, without the barriers of access to face-to-face services, it is anticipated that more youth will have access to a variety of treatments more equitably. However, uptake of and adherence to evidence-based digital interventions as implemented have not been strong to date [11,12].

There are many possible ways to improve the population impact of digital interventions, both in the development of tools and in their delivery. Those developing interventions can enhance user engagement through co-design approaches, in which potential users are actively involved in the development process, ensuring that the products or outputs are optimally placed to meet their needs. Other opportunities include the increased use of telepresence, gamification, and contemporary styles and formats [13]. Implementation processes are also important. For example, promotional activities are needed to ensure that potential users and those who might support them are aware of and interested in digital health options. Embedding and integrating digital mental health tools within existing health or education formats or providing integrated clinical support could also increase uptake and use [13].

### Co-Design

Co-design approaches have been proposed to increase uptake and engagement [14]. In co-design, end users are involved in the development of the intervention through an ongoing democratic partnership with researchers, with a value-based focus on relationships and distribution of power between the *researchers* and the *researched* [15]. Co-design processes can also mean that contextual variables and issues with usability and implementation that may not be obvious to researchers are considered in development. Hence, these processes may increase the acceptability of the end product to the user group. Although there are compelling reasons to involve end users in design, there has been limited evidence to date that consumer involvement in the design of youth mental health interventions improves intervention effectiveness [16]. Furthermore, co-design presents multiple challenges. Co-designers are a subset of the proposed end users and their engagement in the design process can change their preferences. Findings may not generalize to users who were not engaged in development or to those of different ages, cultural groups, and level of need. If end users have diverse or differing needs, there is no guarantee that co-design will enhance impact for all end users. For example, adolescents who are not depressed typically view seeking help and taking action as relatively straightforward, whereas those experiencing depression may find these steps more challenging [17]. Rapidly changing digital technology standards and expectations also mean that findings may not be generalizable over time.

Māori and Pacific peoples form a significant minority of the New Zealand population (approximately 15% and 8%, respectively), are over-represented in higher deprivation settings [18], and are often poorly served by mental health services situated within Western science and health traditions [19]. Māori and Pacific models of mental health and well-being are frequently described as more holistic and family-centered than traditional Western models [19-21]. An explicit focus on underserved communities is important for ensuring relevant services and for supporting equity outcomes.

### Aims

**Figure 1:**
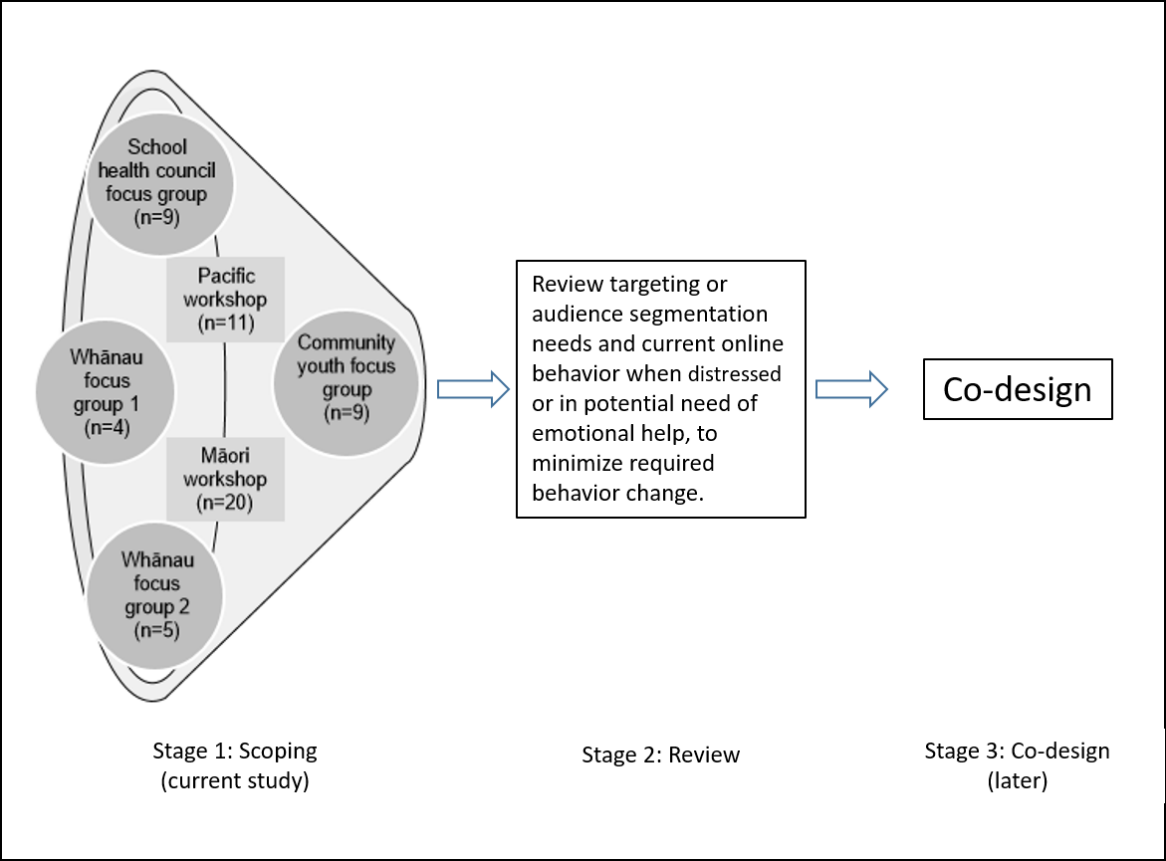
Scoping using diverse processes to inform co-design.

We undertook a preliminary scoping phase to inform a planned co-design process, as shown in Figure 1. Our aim was to explore diverse adolescents’ preferences to understand the need for specific targeting or audience segmentation and key needs of different populations. We also aimed to explore adolescents’ current Web-based behavior when distressed or in potential need of emotional help, to minimize required behavior change.

## Methods

### Overview

The scoping study took place from December 2016 to April 2017. Focus groups in whānau (extended family), school and community groups, as well as interactive workshops, were planned to engage young people in varied ways and encourage a broad spectrum of feedback from the target audience. Ethics approval was obtained from the Health and Disability Ethics Committee (Ref no 16/NTX/174). Young people were included if they were aged between 12 and 25 years, could provide informed consent, and could participate safely. No young people were excluded. We purposively sampled high proportions of Māori and Pacific young people from varied socioeconomic backgrounds and sought to include participants with personal experience of mental distress by recruiting via community groups and networks that included young people with these characteristics.

### Focus Groups

Focus groups of 30- to 60-min duration were held with young people from (1) a school health council, (2) a community youth health organization, and (3) 2 whānau groups. Each whānau focus group included Māori young people who were related to each other, and each took place in a family home. Participants were siblings, cousins, and close family friends aged between 12 and 25 years. Adult family members welcomed the researchers and participants and encouraged participants to take part but were not in the room at the time of the group meeting.

### Interactive Workshops

A total of 2 workshops of approximately 4 hours each were run during school holidays. The first was at a community youth center with an existing youth advisory group, predominantly of Pacific descent. The other was at a Māori health organization site with attendees primarily comprising Māori youth group leaders, members of a youth organization, and school students. These workshops were designed to engage Pacific and Māori youth, respectively, and to encourage their participation.

### Procedures

Participants were asked about their current behavior and needs regarding online mental health tools, their views on currently available online support options, and their preferences regarding pathways to finding the tool, the modality, the use of gamification, and feedback options. They were given tablets loaded with a range of existing mental health apps and websites to prompt discussion. These apps and websites were readily available in New Zealand, were potentially familiar to young people, and displayed a range of styles or ways of providing digital mental health and well-being support. In particular, we included 4 information, treatment, or support resources funded by the New Zealand government (SPARX, The Lowdown, depression.org and Aunty Dee), an Australian mental health resource (Biteback), and several apps available via the App Store (Smiling Mind, Headspace, and SuperBetter).

In each setting, and particularly in the extended workshops, time was allocated to interactive activities such as icebreakers and open discussions using a cultural process that encourages participants to share experiences and stories, an approach commonly used in Pacific and Māori research [22]. This approach takes into consideration relationships that lay the foundation for most Pacific activities [22]. Pacific authors highlight that maintaining good relationships is important for the success of research as they establish trust between researcher and participants. The process further eliminates the distance between the 2, allowing for flexibility in conversation. The Māori workshop followed culturally appropriate protocol. There was a formal welcome of the researcher guests, with an elder opening the session with a prayer, followed by a formal speech. The guests were then invited to introduce themselves and build relationships with the Māori youth. The elder also closed the workshop according to traditional protocols with prayer. The content of the workshops included discussion, sketching ideas on paper, telling stories and *wall storms* where participants wrote ideas on sticky notes, posted them on a wall, then shared and processed them as a group.

At the end of the sessions, participants completed a written summary reporting their age, ethnicity, gender, and whether they had ever felt low or depressed for more than few days in a row. As is customary in Māori and Pacific research, food was provided as part of the process of engaging with people from the community. Each participant was given a voucher as thanks for taking part.

### Data Analysis

Audio recordings from all sessions were transcribed, and facilitators summarized data from sessions that were not recorded due to practicalities (eg, *wall storms* do not lend themselves to audio recording and transcription). Raw data were analyzed and coded in QSR NVivo software. A general inductive approach was used, allowing the main themes to arise from young people’s feedback [23,24]. All transcripts were individually coded among several researchers, including Māori and Pacific coders. First, data were clustered together in categories or groupings. Researchers then convened to discuss potentially important themes and clusters and review the emerging themes against the predefined research questions. This process was repeated several times to ensure data were accurately represented and differing opinions settled. This general inductive approach is an appropriate method for scoping general usability preferences as it highlights information that is relatively exploratory [24].

## Results

### Participants

There were a total of 58 participants drawn from 2 whānau groups (n=4 and n=5), 1 School Health Council group (n=9), 1 Community Youth group (n=9), 1 Pacific workshop (n=11), and 1 Māori workshop (n=20). The majority of participants were female and Māori or Pacific (see Table 1 for demographics). A total of 3 high-level themes emerged.

**Table 1:** Demographic details of study participants (N=58).

Demographics		Focus groups, n				Interactive workshops, n			Total (N=58), n	
		Whānau 1 (n=5)	Whānau 2 (n=4)	School health council (n=9)	Community youth group (n=9)		Pacific (n=11)	Māori (n=20)		
**Gender**										
	Female	3	4	8	7		8	11		41
	Male	2	0	1	2		3	5		13
	Missing	0	0	0	0		0	4		4
**Felt low or depressed for more than a few days in a row**										
	Yes	1	4	6	8		6	11		36
	No	4	0	3	1		5	4		17
	Missing	0	0	0	0		0	5		5
**Ethnicity**										
	Māori	1	2	0	1		1	12		17
	Pacific	3	0	5	1		8	1		18
	NZE^a^	1	2	0	4		1	1		9
	Other	0	0	3	1		1	1		6
	Missing	0	0	1	2		0	5		8
**Age (years)**										
	<14	1	0	1	0		0	0		2
	14-17	0	4	8	1		8	5		26
	>17	4	0	0	8		3	10		25
	Missing	0	0	0	0		0	5		5

^a^NZE: New Zealand European.

### Theme 1: Digital Mental Health Tools are Unlikely to be Successful if They Rely on Youth Help-Seeking

Respondents described a range of strategies they used to deal with distress. Social withdrawal, using drugs and alcohol, and self-harm were common. Other responses included ignoring the distress or using music, prayer, sport, or other activities as a distraction. Most expressed a preference for “keeping personal stuff” to themselves and coping on their own, for example:

I don't really tell anyone or people about what I'm going through.

School Health Council

People probably don’t say anything, just keep it to themselves.

Whānau group 1

Participants were concerned and embarrassed about others knowing they were “having a hard time.” Talking distress over with friends and/or family and/or seeking help from a known adult (such as a trusted teacher) were relatively rare, and no participants mentioned seeking help from unknown adults. Despite this, some participants reported that they would post about their distress on social media (such as Facebook or Instagram), on a personal account or anonymously, in the hope that others might understand or reach out to them. For example:

You have 8 different accounts and nobody knows who you are. So there’s no stigma attached to saying anything. You can just be truthful.

Community youth group

Like on Facebook, you can do like feeling, you can put up an emotion to what you’re feeling. So people would be like sad face- feeling down or something…

Whānau group 1

Aside from possible posts on social media, no participants spontaneously mentioned that they would seek help or use the internet for mental health needs. They reported that they would feel labeled and stigmatized after accessing mental health websites even if nobody knew:

Yeah, you’d feel embarrassed [accessing a mental health website].

Whānau group 1

In response to direct questions from the researchers, participants reported that they would seek help only if they were “desperate,” in which case their preference may be posting on social media where friends might respond.

Despite this lack of active help-seeking, participants responded positively to the idea of online support:

I think it sounds like a great idea because it’s on mobile and a lot of people are getting phones and stuff…everyone, like especially the teenagers, they’re always on their phone…So it’s cool to have it on their phones.

School Health Council

...the power that comes from an app is the fact that apps have push notifications and stuff like that, that remind you [to] engage with them…an app would be a good place to have your more interactive parts, and then a website would be a good place to have facts and information and where you can seek help and stuff…

Community youth group

These findings were considered alongside the imperative to require realistic behavior change for the use of any digital mental health app or tool. The theme of *Digital mental health tools are unlikely to be successful if they rely on youth help-seeking* was articulated and confirmed by rereading transcripts, reviewing original codes, and discussion among researchers.

### Theme 2: A Single Approach is Unlikely to Appeal to All

Importantly, different groups and individuals had diverse, noncompatible preferences in terms of *look or feel* of an app or digital tool. Being relevant and appealing for young people was considered key; pitching information in the right way also mattered. However, what was *the right way* differed among participants. Many young people preferred a crisp, clean look, which was uncomplicated with straightforward, direct, clear, and “straight-to-the-point” help that they would access when they or others were in a crisis. They considered they would not normally look for this kind of help unless they were very worried about someone or were suicidal. In this context, they prioritized relatively serious content; some felt that a game or narrative approach would be off-putting or inappropriate. This view was more frequently expressed by older participants and those who indicated that they had had some experience of distress:

That person has literally hit the point where they can’t reach out anymore. They need something that engages with them instantly [rather than is playful or indirect].

Community youth group

If it was like gaming aspects in there, like characters, like moving up a level or something, [it’s not right] because it is quite a serious matter. So yeah, take it seriously.

School Health Council

I’d go straight to the point kind of stuff. Like I’d want to know bullet point, then another bullet point.

Whānau group 1

In contrast, other participants considered that a *gamey*, fun, or entertaining environment could engage people who might not otherwise recognize that they need help or be seeking help. A nonthreatening, normalizing, nonpathologizing approach was seen as key, and participants suggested focusing on identity, everyday problems, well-being, strengths, and inspiration. These were often younger participants with less experience of mental distress:

I do like the idea of a game. Yeah, that is a cool idea.

Whānau group 2

Like it’s actually engaging but it’s also teaching you stuff.

Whānau group 2

And yeah, try and make it as a game. Yeah, ‘cos it’s fun.

School Health Council

These findings were considered alongside the research needs. The theme of *a single approach unlikely to appeal to all* was articulated and confirmed by rereading transcripts and reviewing original codes. Next, we clustered user preferences. A total of 4 major groupings or categories emerged:

1. Those who were more interested in a digital intervention to support their mental well-being if it was *fun* to do. Many reported that they would engage in an intervention that was storified or gamified. These participants were often younger adolescents who were not feeling stressed out or down at the time of the study. We identified them as *Players or gamers.*
2. Adolescents who were interested in digital interventions that were targeted directly to support their mental well-being, interactive and potentially gamified. Again, these participants were often younger adolescents, but they generally had more experience of feeling stressed or down than *Players*. We categorized them as *Engagers.*
3. Young people who were not personally interested in digital interventions for mental health. These participants felt no need for such interventions and were skeptical about their value, expressing more interest in social media and interacting with their friends. This group generally comprised older adolescents who were not feeling stressed out or down at the time of the study. We identified this group as *Skeptics*.
4. Finally, a cluster of young people expressed interest in *straight to the point* digital interventions for mental well-being. They considered gamification off-putting and indicated a preference for something simple with a clean, clear design that conveyed the message *straight up*. This cluster mainly comprised older adolescents experiencing stress or low mood. We categorized this group as *Straight-talkers*.

**Figure 2:**
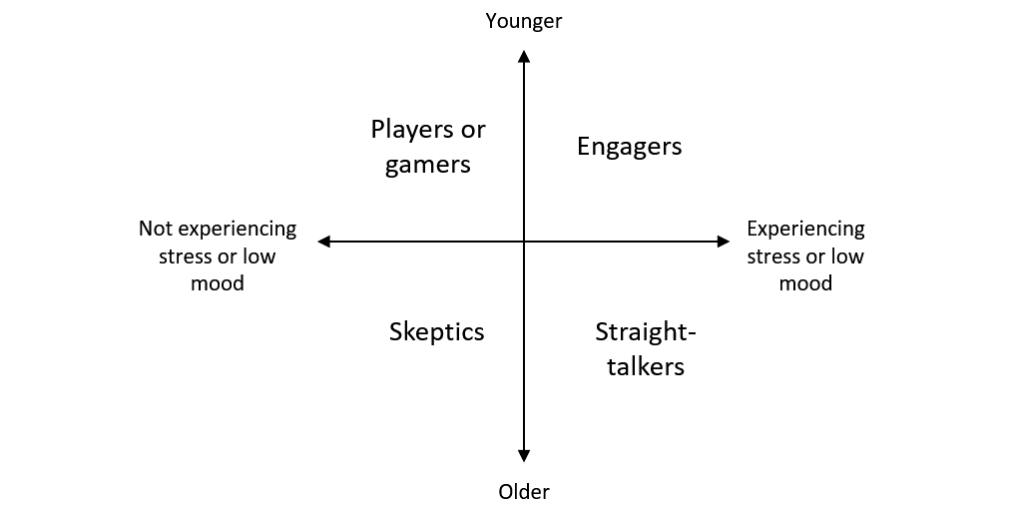
Taxonomy of adolescents engaging with digital mental health tools.

We proposed that these clusters can be represented in a taxonomy as shown in Figure 2.

### Theme 3: Help Here and Now, With Multiple Challenges

Despite differences in preferred style, there was a high level of consensus that young people preferred content that made them feel good and helped them cope with hard times, with impact in the *here and now* rather than in the future. Participants also reported that they wanted freedom of choice, including the ability to pick and choose interventions and would want help with a variety of challenges or needs such as bullying, relationship problems, self-harm, suicide, and other mental health needs:

Today [it] is all about choice, really.

Community youth group

Yeah I think apps that had different components, so maybe like a combination of meditation and information or interactive kind of things

Whānau group 2

## Discussion

### Principal Findings

Participants reported that they did not actively seek help if down or distressed unless they were very concerned, for example, if there might be a risk of suicide. If they did use the internet when feeling down or distressed, this consisted of posting on a social media site where friends might understand or respond. Despite this lack of active help seeking, participants responded positively to the idea of online support. There were diverse preferences for the look and style of such interventions. Older adolescents and those experiencing distress were interested in direct, to-the-point information and considered that gamification might be trivializing. They wanted tools that connected to social support in some way. Some, especially younger, participants were more interested in game-like and playful approaches that might have a more universal appeal and were less directly mental health–focused. These differences in preferences highlight the challenge in trying to *be all things to all people* and the need for targeted approaches or *audience segmentation* in co-design and marketing terms. Finally, participants highlighted the need for immediate help with multiple challenges.

### Comparisons With Previous Work

These results suggest that improving youth mental health via specific digital interventions has promise. However, acknowledging young people’s lack of help-seeking and diverse preferences is critical to the successful implementation of digital mental health interventions.

Our finding that participants were unlikely to seek help for mental health needs online is consistent with previous literature [25]. There are major challenges reaching groups that have low rates of help-seeking if approaches rely on users proactively and individually seeking help. Young people are generally poor at seeking help for emotional distress for myriad reasons including time limitations, privacy concerns, financial reasons, and skepticism about treatment effectiveness [26]. Unsurprisingly, those from high deprivation neighborhoods find it hardest to access health care [27]. To reach those with high needs, online interventions may need to be promoted or actively introduced, for example, through routine incorporation into established systems that young people are exposed to, such as school health checks or the school health curriculum. For those not attending school or where a school-based approach is less likely to hold appeal, an approach that utilizes commonly used media may be more appropriate. For example, many young people currently use *Messenger*; a chat-bot using this conversational channel may make an intervention more appealing and accessible. Previous research has highlighted that young people do look for health information online [28,29]. Hence, search engine advertisements and websites providing information could be used to help encourage young people to seek help, whether online or face-to-face.

Our finding that participants had diverse and often conflicting preferences, even within this relatively specific demographic, shows that efforts to deliver digital interventions risk failure if developers try to be all things to all people. In fact, if we were to *average* all results, we might be inclined to suggest that an intervention needed to be *a little bit playful*, whereas in reality, results were dichotomous. This finding should come as no surprise as a similar phenomenon is apparent among those accessing the internet for other reasons. For example, Bartle, long ago proposed a taxonomy of *gamers* with differing views about what appeals in computer games [30]. He proposed 4 categories of gamers: killers, explorers, socializers, and collectors. This idea has since been expanded [31]. In many current games, one can see specific designs to appeal to the varying populations of likely users. Our finding of broad clusters of users and our proposed taxonomy of young people who use digital interventions to support their mental health is consistent with these concepts of audience segmentation.

Such analyses can be used to inform the design of digital interventions to appeal to specific groups. For example, interventions that aim to prevent emotional health problems might be delivered to young groups using gamified or storified approaches. Designing for young people who are seeking help for established problems might require a more direct approach.

### Implications

The results of this study suggest that online approaches to improve the mental health of young people, particularly Māori and Pacific young people in New Zealand, should consider the following needs:

- Direct and clear information and immediate access to support for those who are seeking help in a crisis;
- Interventions targeting those posting about their distress on social media (eg, this can include social media platforms providing direct-to-user messages or alerts to services, based on user-generated content);
- Approaches that engage young people before their needs are high and before they would be seeking help in current contexts. This might be done via health promotion and marketing to attempt to increase interest and reduce stigma [32], increasing the attractiveness and appeal of interventions, or mandated systems that target groups are exposed to (eg, universal screening with interventions via health or education systems).

The wide range of opinions reported by our participants highlights that digital interventions that aim to appeal to all young people risk failure and that more targeted strategies may have more promise. Should future research support our taxonomy of Players or gamers, Engagers, Skeptics, and Straight-talkers, we should expect a range of youth-oriented mental health tools, with some serious and direct and others more playful or gamified. Segmentation requires a greater investment of resources than a single approach that reaches all. In other areas, however, from games to news sites, online tools are diverse, a feature considered essential for engagement. Indeed, a single approach that aims to reach all and in doing so appeals to none or few cannot be classed a success. Increased diversity and targeting of digital mental health options should be explored as an approach to improve engagement.

The identification of conflicting user preferences also highlights the value of scoping user needs and preferences *before* establishing co-design processes. For example, without identifying varied needs, we may have developed a mixed group of co-designers pulling in competing directions or co-operating to produce an outcome not particularly appealing to any.

Finally, certain characteristics appear important across all groups, including the provision of choice. Young people wanted to be able to pick and choose those interventions that suited them. In the same way that providing a *menu of options* is an important component of brief face-to-face interventions, it will be important for digital interventions to provide users with the experience of being in control of the process [33].

### Limitations

This is a small and very specific sample. We used a purposive sample that included indigenous Māori and Pacific youth who provided information about their experiences of digital resources; few studies exist that have sought to capture this information. Although useful in providing needed information about this group, our focus on Māori and Pacific youth in New Zealand and small group sizes limits the generalizability of findings to other demographics. Future research should scope diversity of user preferences within other population and health-need groups.

### Conclusions

This study demonstrates the importance of scoping and consulting before designing. Youth have varied preferences and it is essential to decide on a focused approach and then co-design with adolescents in a particular group if engagement and implementation are to succeed. The need for varied interfaces is clear.

The use of technology to increase the delivery of psychological interventions to support the mental health of young people remains a promising approach, but successful implementation will require a greater degree of sophistication in design and rollout than has been demonstrated to date.

## Abbreviations

HABITs: Health Advances through Behavioural Intervention Technology
NZE: New Zealand European
SPARX: Smart Positive and Realistic X-Factor Thoughts

## References

[1] Teevale T, Lee AC, Tiatia-Seath J, Clark TC, Denny S, Bullen P, Fleming T, Peiris-John RJ (2016). Risk and protective factors for suicidal behaviors among Pacific youth in New Zealand. Crisis.

[2] Clark TC, Robinson E, Crengle S, Fleming T, Ameratunga S, Denny SJ, Bearinger LH, Sieving RE, Saewyc E (2011). Risk and protective factors for suicide attempt among Indigenous Māori Youth in New Zealand: the role of family connection. J Aborig Health.

[3] Peni B, Day K, Orr M (2014). What Pacific people think of online mental health information. Proceedings of the Seventh Australasian Workshop on Health Informatics and Knowledge Management.

[4] Denny S, Farrant B, Cosgriff J, Harte M, Cameron T, Johnson R, McNair V, Utter J, Crengle S, Fleming T, Ameratunga S, Sheridan J, Robinson E (2013). Forgone health care among secondary school students in New Zealand. J Prim Health Care.

[5] Mariu KR, Merry SN, Robinson EM, Watson PD (2012). Seeking professional help for mental health problems, among New Zealand secondary school students. Clin Child Psychol Psychiatry.

[6] Stasiak K, Fleming T, Lucassen MF, Shepherd MJ, Whittaker R, Merry SN (2016). Computer-based and online therapy for depression and anxiety in children and adolescents. J Child Adolesc Psychopharmacol.

[7] Ebert DD, Zarski A, Christensen H, Stikkelbroek Y, Cuijpers P, Berking M, Riper H (2015). Internet and computer-based cognitive behavioral therapy for anxiety and depression in youth: a meta-analysis of randomized controlled outcome trials. PLoS One.

[8] Rajagopalan A, Shah P, Zhang MW, Ho RC (2017). Digital platforms in the assessment and monitoring of patients with bipolar disorder. Brain Sci.

[9] Shepherd M, Fleming T, Lucassen M, Stasiak K, Lambie I, Merry SN (2015). The design and relevance of a computerized gamified depression therapy program for indigenous Māori adolescents. JMIR Serious Games.

[10] Fleming T, Dixon R, Frampton C, Merry S (2012). A pragmatic randomized controlled trial of computerized CBT (SPARX) for symptoms of depression among adolescents excluded from mainstream education. Behav Cogn Psychother.

[11] Loiselle CG, Ahmed S (2017). Is connected health contributing to a healthier population?. J Med Internet Res.

[12] Fleming T, Bavin L, Lucassen M, Stasiak K, Hopkins S, Merry S (2018). Beyond the trial: systematic review of real-world uptake and engagement with digital self-help interventions for depression, low mood, or anxiety. J Med Internet Res.

[13] Fleming TM, de Beurs D, Khazaal Y, Gaggioli A, Riva G, Botella C, Baños RM, Aschieri F, Bavin LM, Kleiboer A, Merry S, Lau HM, Riper H (2016). Maximizing the impact of e-therapy and serious gaming: time for a paradigm shift. Front Psychiatry.

[14] Hagen P, Collin P, Metcalf A, Nicholas M, Rahilly K, Swainston N (2012). Young and Well Cooperative Research Centre.

[15] Goodyear-Smith F (2017). Collective enquiry and reflective action in research: towards a clarification of the terminology. Fam Pract.

[16] Orlowski SK, Lawn S, Venning A, Winsall M, Jones GM, Wyld K, Damarell RA, Antezana G, Schrader G, Smith D, Collin P, Bidargaddi N (2015). Participatory research as one piece of the puzzle: a systematic review of consumer involvement in design of technology-based youth mental health and well-being interventions. JMIR Hum Factors.

[17] Wilson CJ, Deane FP (2010). Help-negation and suicidal ideation: the role of depression, anxiety and hopelessness. J Youth Adolesc.

[18] Atkinson J, Salmond C, Crampton P (2014). Department of Public Health, University of Otago.

[19] Durie M (2001). Maori Ora: The Dynamics of Māori Health.

[20] Pere R (1982). Concepts and Learning in the Māori Tradition.

[21] Tamasese K, Peteru C, Waldegrave C, Bush A (2016). Ole Taeao Afua, the new morning: a qualitative investigation into Samoan perspectives on mental health and culturally appropriate services. Aust NZ J Psychiatry.

[22] Vaioleti TM (2006). Talanoa research methodology: a developing position on Pacific research. WJE.

[23] Braun V, Clarke V (2006). Using thematic analysis in psychology. Qual Res Psychol.

[24] Thomas DR (2006). A general Inductive approach for analyzing qualitative evaluation data. Am J Med Eval.

[25] Utter J, Lucassen M, Denny S, Fleming T, Peiris-John R, Clark T (2017). Using the Internet to access health-related information: results from a nationally representative sample of New Zealand secondary school students. Int J Adolesc Med Health.

[26] Hunt J, Eisenberg D (2010). Mental health problems and help-seeking behavior among college students. J Adolesc Health.

[27] Clark T, Fleming T, Bullen P, Denny S, Crengle S, Dyson B, Fortune S, Lucassen M, Peiris-John R, Robinson E (2013). The University of Auckland.

[28] Utter J, Lucassen M, Denny S, Fleming T, Peiris-John R, Clark T (2017). Using the internet to access health-related information: results from a nationally representative sample of New Zealand secondary school students. Int J Adolesc Med Health.

[29] Wartella E, Rideout V, Montague H, Beaudoin-Ryan L, Lauricella A (2016). Teens, health and technology: a national survey. Media Communication.

[30] Bartle R MUD.

[31] Stewart B Gamasutra.

[32] Clement S, Lassman F, Barley E, Evans-Lacko S, Williams P, Yamaguchi S, Slade M, Rüsch N, Thornicroft G (2013). Mass media interventions for reducing mental health-related stigma. Cochrane Database Syst Rev.

[33] (2001). Brief intervention for hazardous and harmful drinking : a manual for use in primary care.

